# Supplementary material for: Isoflavone Consumption and Risk of Breast Cancer: An Updated Systematic Review with Meta-Analysis of Observational Studies
Source: Nutrients. 2023 May 21;15(10):2402. doi: 10.3390/nu15102402 (PMC10224089; doi:10.3390/nu15102402)
Supplement: Supplementary file 1 [file nutrients-15-02402-s001.zip › Figure S1 A.pdf]

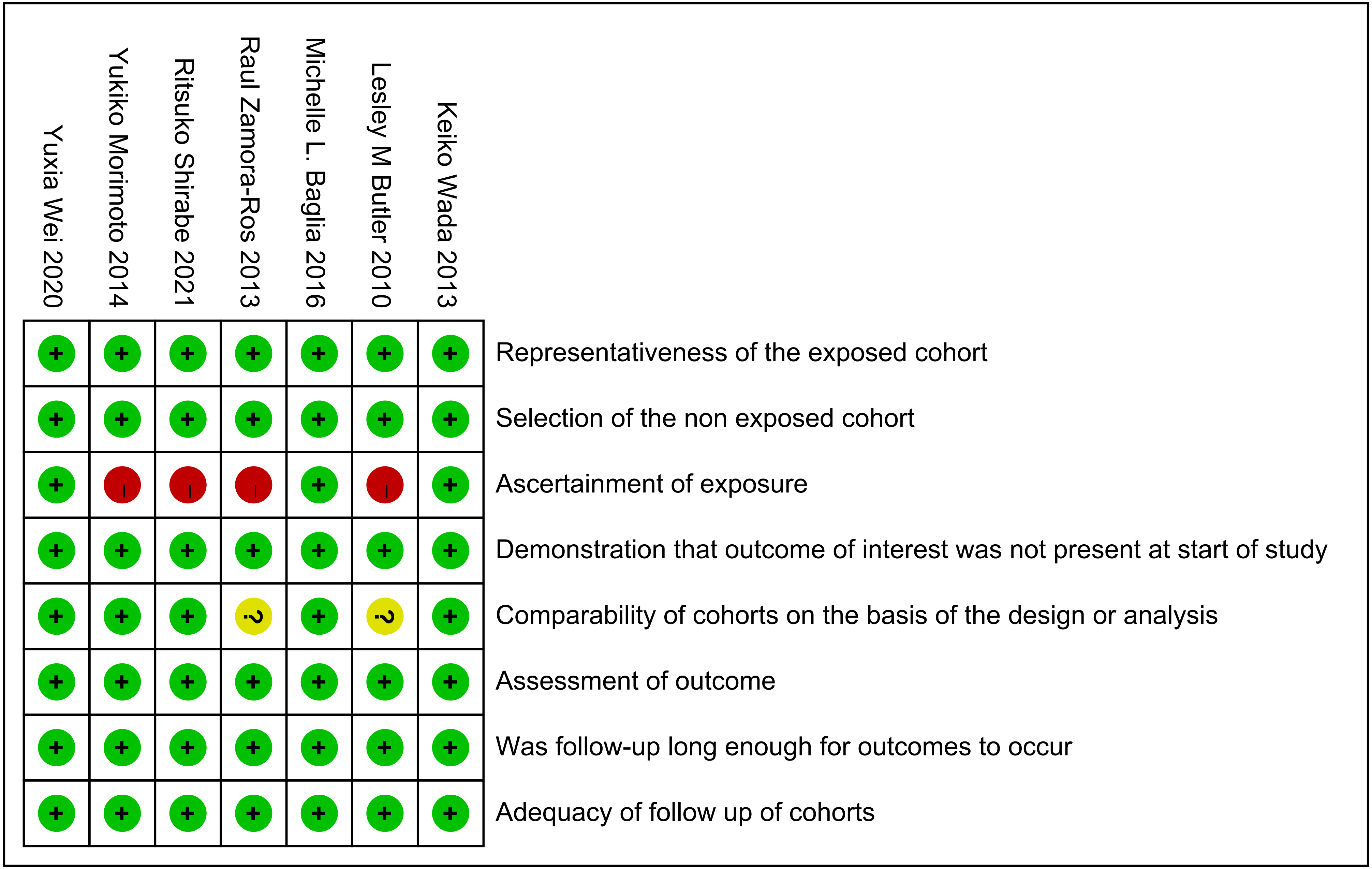
Yuxia Wei 2020
Yukiko Morimoto 2014
Ritsuko Shirabe 2021
Raul Zamora-Ros 2013
Michelle L. Baglia 2016
Lesley M Butler 2010
Keiko Wada 2013
Representativeness of the exposed cohort
Selection of the non exposed cohort
Ascertainment of exposure
Demonstration that outcome of interest was not present at start of study
Comparability of cohorts on the basis of the design or analysis
Assessment of outcome
Was follow-up long enough for outcomes to occur
Adequacy of follow up of cohorts
