# Supplementary material for: Isoflavone Consumption and Risk of Breast Cancer: An Updated Systematic Review with Meta-Analysis of Observational Studies
Source: Nutrients. 2023 May 21;15(10):2402. doi: 10.3390/nu15102402 (PMC10224089; doi:10.3390/nu15102402)
Supplement: Supplementary file 1 [file nutrients-15-02402-s001.zip › Figure S1 B.pdf]

Study	Is the case definition adequate?	Representativeness of the cases	Selection of Controls	Definition of Controls	Comparability of cases and controls on the basis of the design or analysis	Ascertainment of exposure	Same method of ascertainment for cases and controls	Non-Response rate
Yao-Jen Chang 2017	-	+	-	+	+	+	+	-
Yan-yun Zhu 2011	+	+	-	+	+	+	+	+
YA Cho 2010	+	+	-	+	+	+	+	-
Xiao-Li Feng 2019	+	+	-	+	+	+	+	+
Qiong Wang 2011	+	+	+	+	+	+	+	-
Pamela L. Horn-Ross 2001	-	+	-	-	+	+	+	+
Motoki Iwasaki 2008	+	+	-	+	+	+	+	+
Min Zhang 2009	+	+	-	+	+	-	+	+
Michelle Cotterchio 2007	+	+	+	+	+	+	+	-
Masakazu Toi 2013	+	+	+	-	+	+	+	-
Lin Li 2013	+	+	+	+	+	+	+	+
Laura N. Anderson 2012	-	+	+	-	+	+	+	+
K Hirose 2005	+	+	-	+	+	+	+	-
Jakob Linseisen 2004	-	+	+	-	+	+	+	-
Isabel dos Santos Silva 2004	-	+	+	-	+	+	+	+
Caixia Zhang 2009	+	+	-	+	+	+	+	+
Anna H Wu 2002	+	+	+	+	+	+	+	-
