# Supplementary material for: Isoflavone Consumption and Risk of Breast Cancer: An Updated Systematic Review with Meta-Analysis of Observational Studies
Source: Nutrients. 2023 May 21;15(10):2402. doi: 10.3390/nu15102402 (PMC10224089; doi:10.3390/nu15102402)
Supplement: Supplementary file 1 [file nutrients-15-02402-s001.zip › Table S1.pdf]

Table S1.

Description of population, intervention/exposure, comparison, and outcome (PICO).

**Population**	Inclusion: Female adults. Exclusion: Female children or adolescents (under 18 years of age) and male.
**Intervention/Exposure**	Dietary isoflavone are defined as those taken from food/diet, excluding the isoflavone supplements in the form of capsules or tablets, etc.
**Comparison**	The dietary isoflavone consumption (the lowest vs. the highest)
**Outcome**	Breast cancer.
