# Supplementary material for: Isoflavone Consumption and Risk of Breast Cancer: An Updated Systematic Review with Meta-Analysis of Observational Studies
Source: Nutrients. 2023 May 21;15(10):2402. doi: 10.3390/nu15102402 (PMC10224089; doi:10.3390/nu15102402)
Supplement: Supplementary file 1 [file nutrients-15-02402-s001.zip › Table S2.pdf]

Table S2.

Search strategies including the key terms and the queries for each database

Database (1/2000-8/2021)	Key terms and the queries
Pubmed	((("breast cancer"[All Fields] OR "breast carcinoma"[All Fields] OR "breast neoplasm"[All Fields] OR "breast tumor"[All Fields]) AND ("isoflavon"[All Fields] OR "isoflavones"[MeSH Terms] OR "isoflavones"[All Fields] OR "isoflavone"[All Fields] OR "isoflavonic"[All Fields] OR "isoflavons"[All Fields] OR ("genistein"[MeSH Terms] OR "genistein"[All Fields] OR "genistein s"[All Fields] OR "genisteine"[All Fields] OR "genisteins"[All Fields]) OR ("daidzein"[Supplementary Concept] OR "daidzein"[All Fields] OR "daidzein s"[All Fields]) OR ("glycitein"[Supplementary Concept] OR "glycitein"[All Fields]) OR "biochanin"[All Fields] OR ("formononetin"[Supplementary Concept] OR "formononetin"[All Fields]) OR "soy"[All Fields] OR "red clover"[All Fields] OR ("soy foods"[MeSH Terms] OR ("soy"[All Fields] AND "foods"[All Fields]) OR "soy foods"[All Fields] OR "tofu"[All Fields]) OR ("soybeans"[MeSH Terms] OR "soybeans"[All Fields] OR "soya"[All Fields]))) NOT ("cell"[Title] OR "mice"[Title] OR "rats"[Title])) AND 2000/01/01:2021/8/1[Date - Publication]
Web of science	(((TS=(isoflavones or genistein or daidzein or glycitein or biochanin or formononetin or soy or “red clover”)) AND TS=(“breast cancer” OR “breast carcinoma” OR “breast neoplasm” OR “breast tumor”))) NOT TI=(cell OR mice OR rats)
Embase	(isoflavones OR genistein OR daidzein OR glycitein OR biochanin OR form ononetin OR soy OR 'red clover' OR tofu OR soya) AND ('breast cancer' OR 'breast carcinoma' OR 'breast neoplasm' OR 'breast tumor') NOT (cell:ti OR mice:ti OR rats:ti) AND [2000-2021]/py #3 AND ('article'/it OR 'chapter'/it OR 'conference paper'/it OR 'conference review'/it OR 'editorial'/it OR 'erratum'/it OR 'letter'/it OR 'review'/it OR 'short survey'/it OR 'tombstone'/it)
