# Supplementary figures and images for: Isoflavone Consumption and Risk of Breast Cancer: An Updated Systematic Review with Meta-Analysis of Observational Studies
Source: Nutrients. 2023 May 21;15(10):2402. doi: 10.3390/nu15102402 (PMC10224089; doi:10.3390/nu15102402)

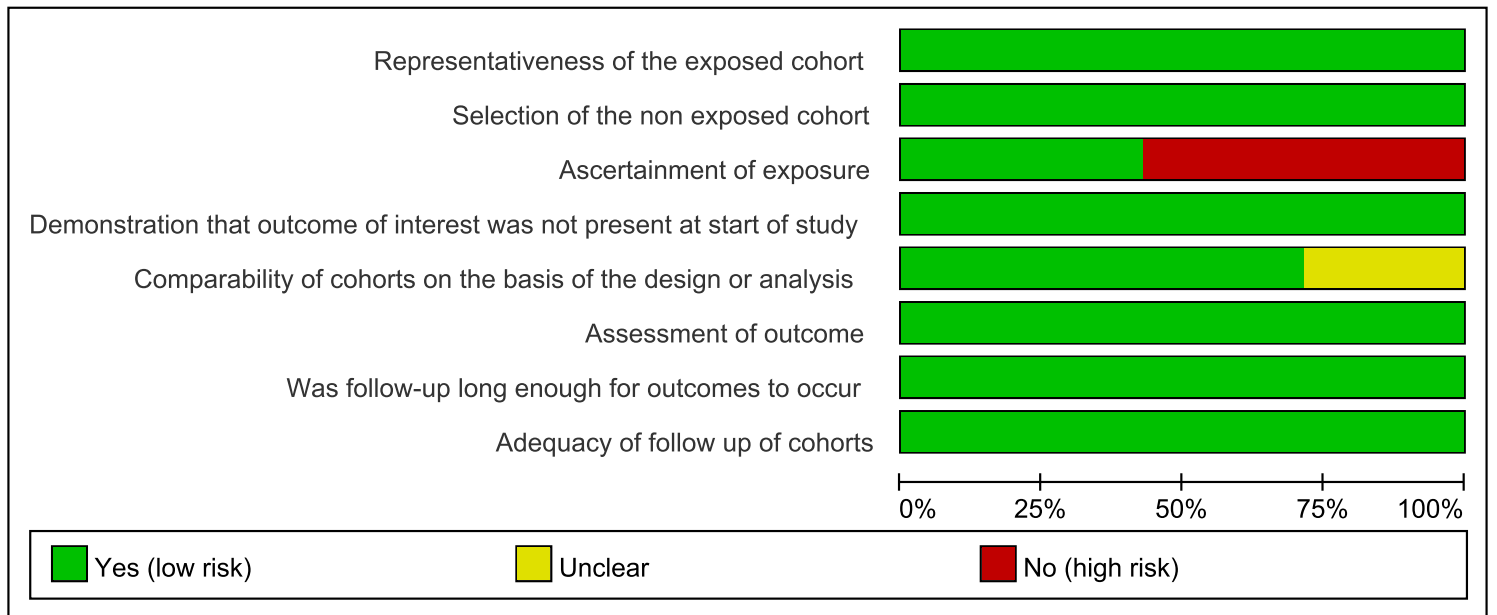

Supplement: Supplementary file 1 [file nutrients-15-02402-s001.zip › Figure S2 A.pdf]

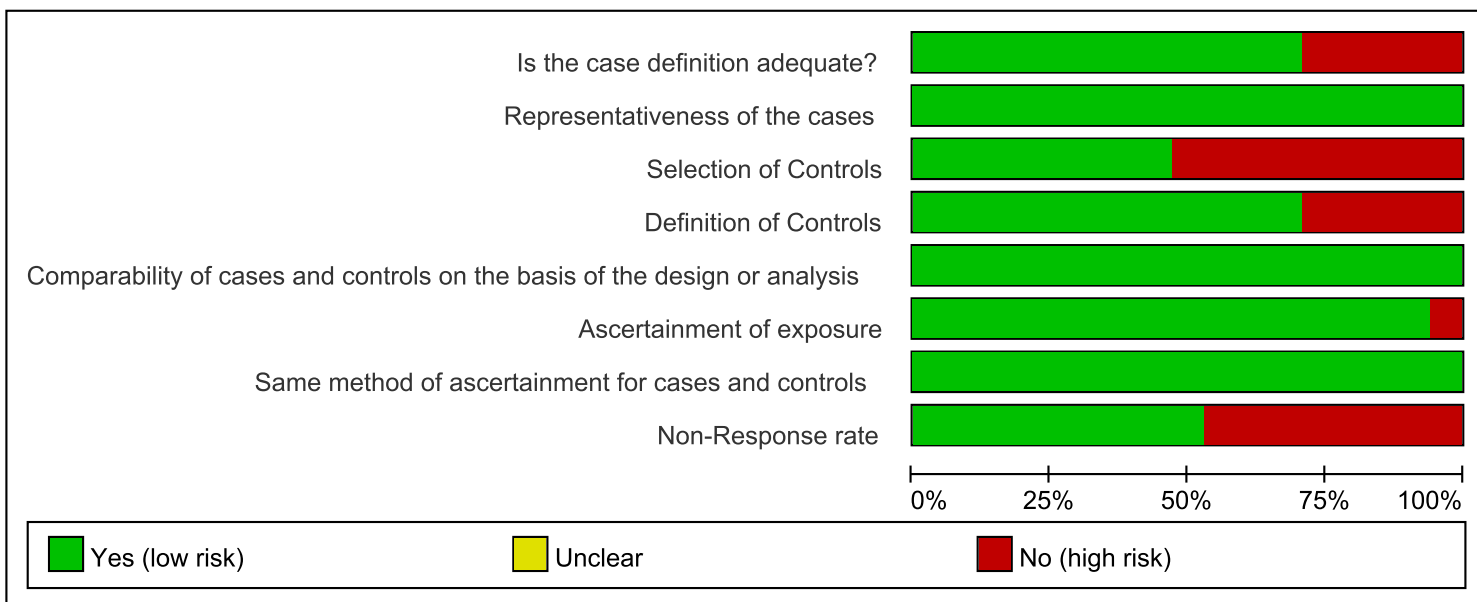

Supplement: Supplementary file 1 [file nutrients-15-02402-s001.zip › Figure S2 B.pdf]

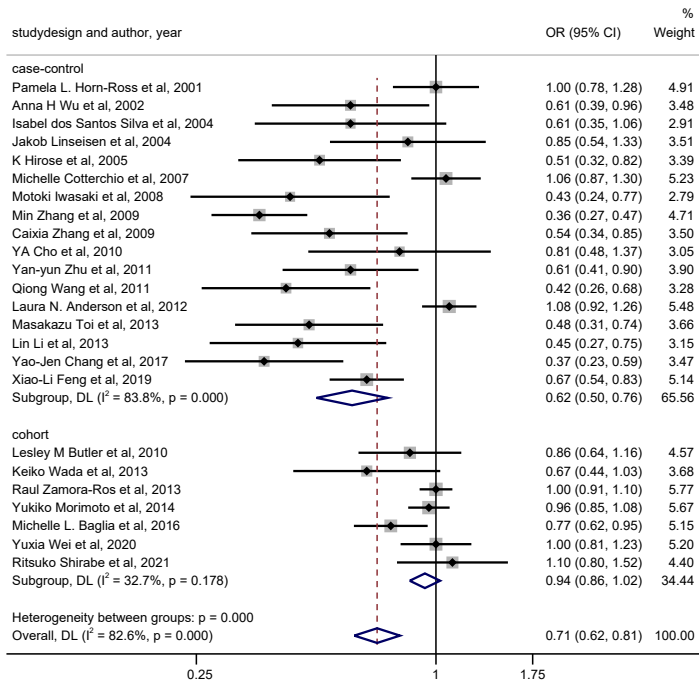

NOTE: Weights and between-subgroup heterogeneity test are from random-effects model

Supplement: Supplementary file 1 [file nutrients-15-02402-s001.zip › Figure S3 A.pdf]

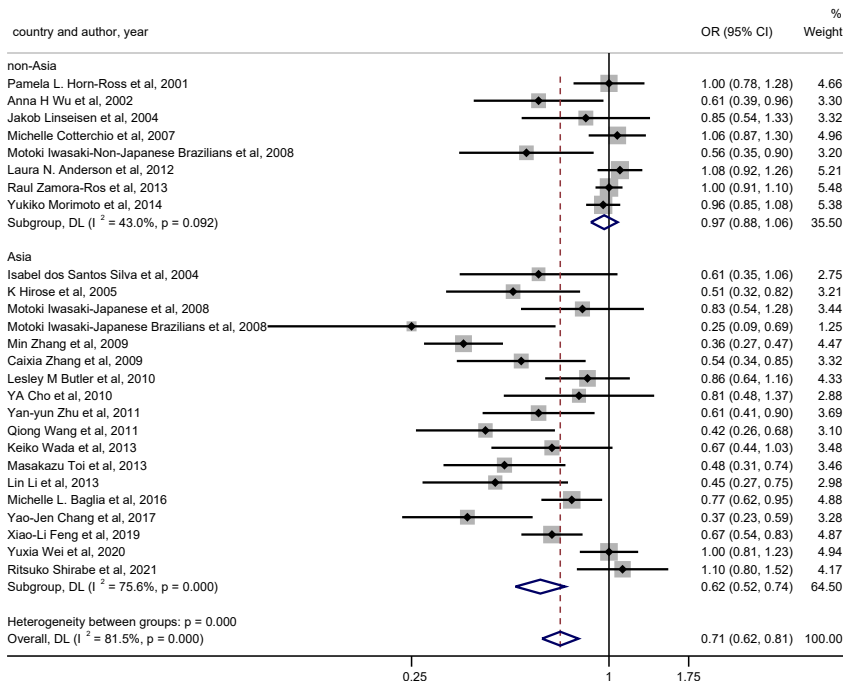

NOTE: Weights and between-subgroup heterogeneity test are from random-effects model

Supplement: Supplementary file 1 [file nutrients-15-02402-s001.zip › Figure S3 B.pdf]

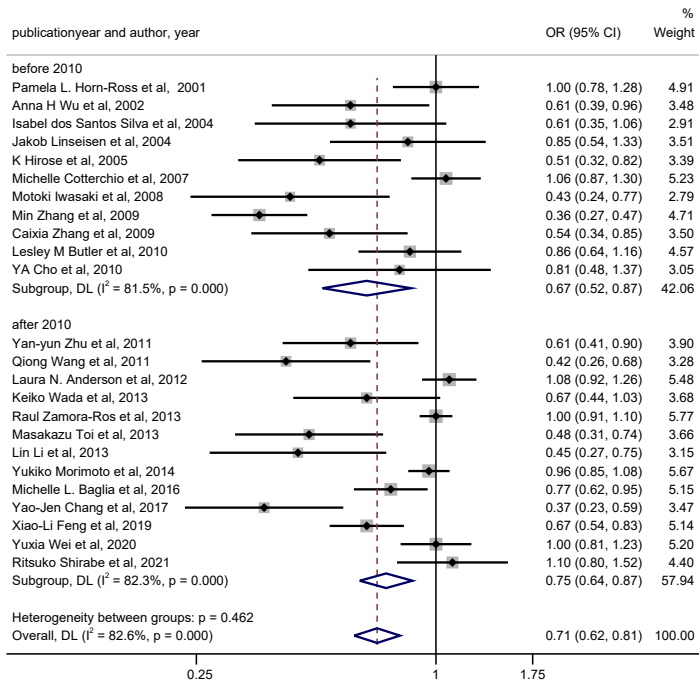

NOTE: Weights and between-subgroup heterogeneity test are from random-effects model

Supplement: Supplementary file 1 [file nutrients-15-02402-s001.zip › Figure S3 C.pdf]

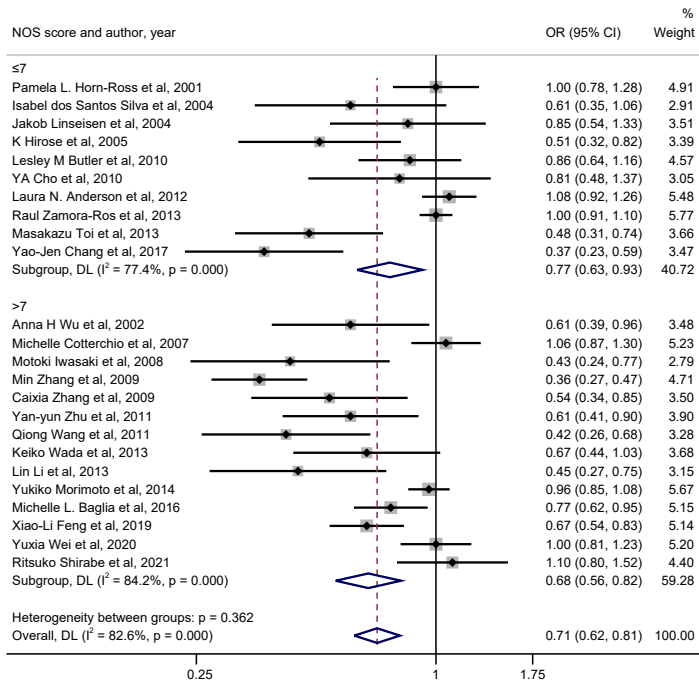

NOTE: Weights and between-subgroup heterogeneity test are from random-effects model

Supplement: Supplementary file 1 [file nutrients-15-02402-s001.zip › Figure S3 D.pdf]

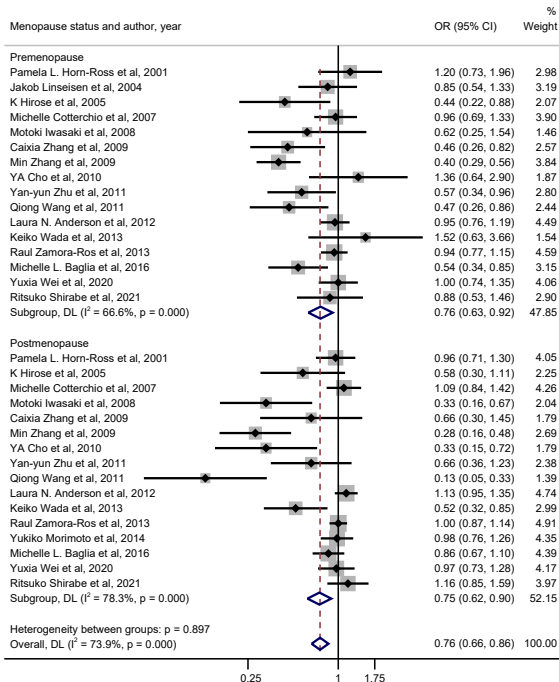

NOTE: Weights and between-subgroup heterogeneity test are from random-effects model

Supplement: Supplementary file 1 [file nutrients-15-02402-s001.zip › Figure S3 E.pdf]

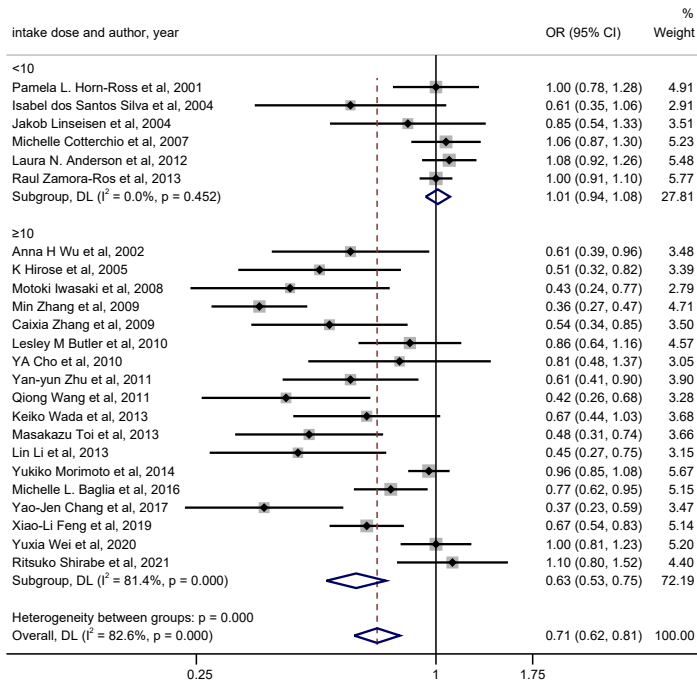

NOTE: Weights and between-subgroup heterogeneity test are from random-effects model

Supplement: Supplementary file 1 [file nutrients-15-02402-s001.zip › Figure S3 F.pdf]

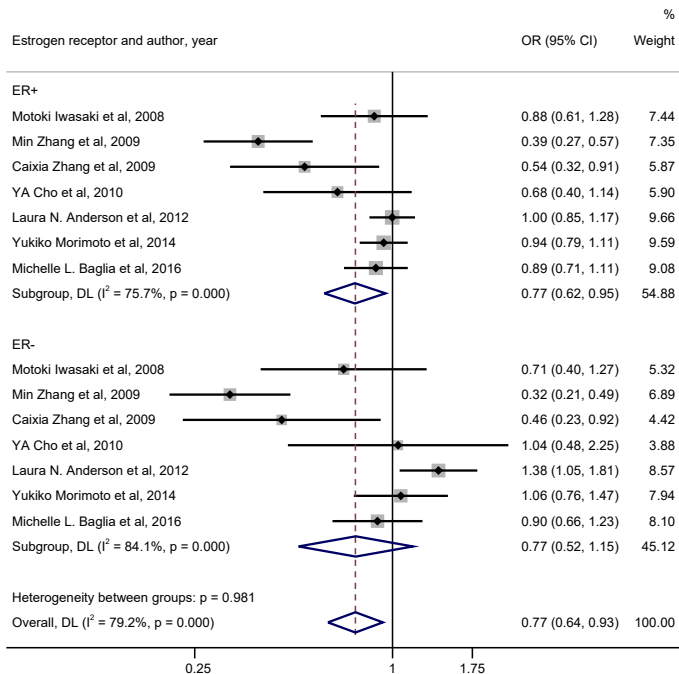

NOTE: Weights and between-subgroup heterogeneity test are from random-effects model

Supplement: Supplementary file 1 [file nutrients-15-02402-s001.zip › Figure S3 G.pdf]

Funnel plot with pseudo 95% confidence limits

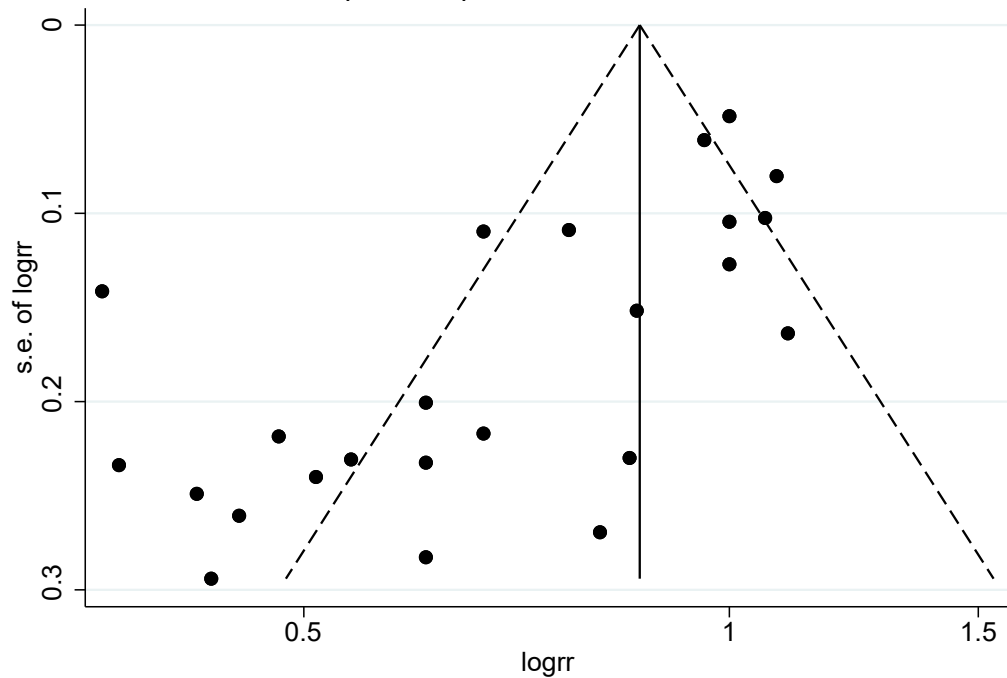

Supplement: Supplementary file 1 [file nutrients-15-02402-s001.zip › Figure S4 A.pdf]

Egger's publication bias plot

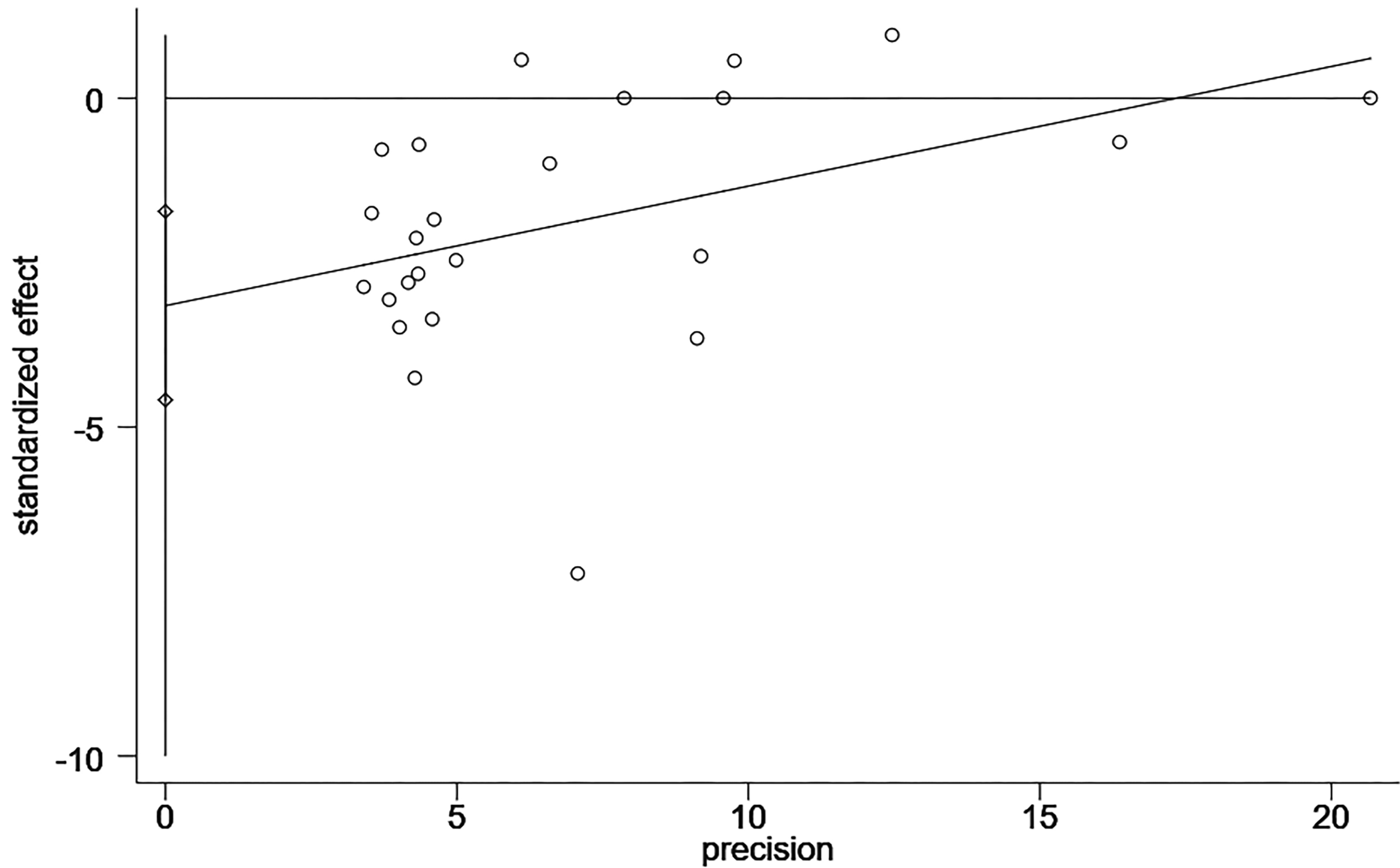

Supplement: Supplementary file 1 [file nutrients-15-02402-s001.zip › Figure S4 B.pdf]

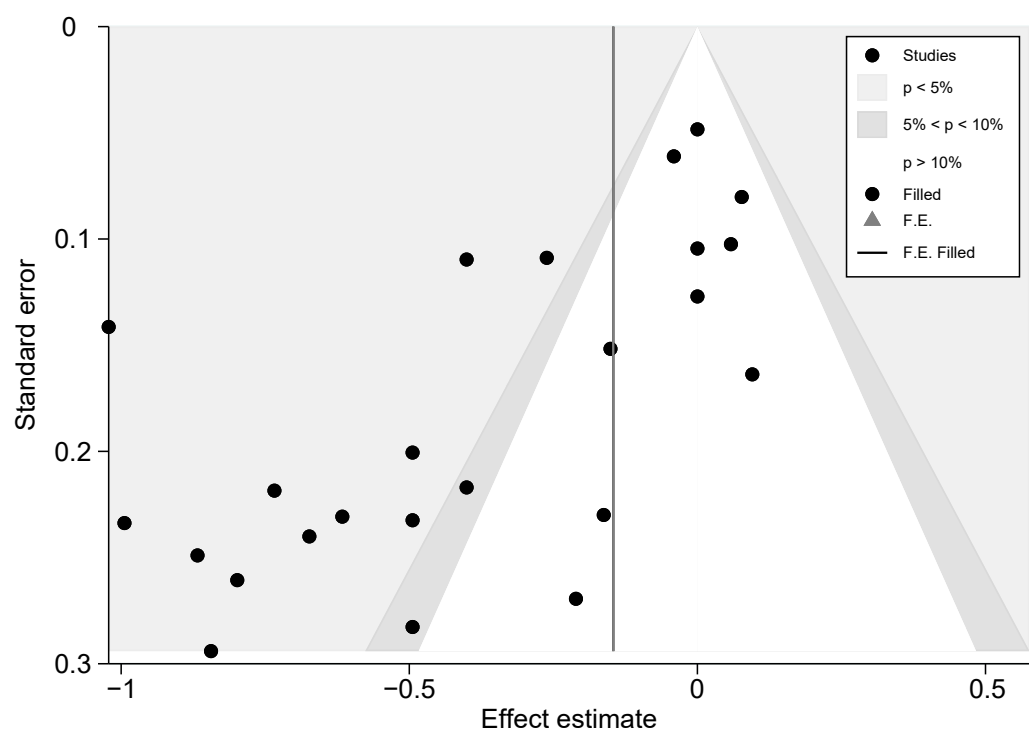

Supplement: Supplementary file 1 [file nutrients-15-02402-s001.zip › Figure S4 C.pdf]

# Meta-analysis estimates, given named study is omitted

| Lower CI Limit    ○ Estimate    | Upper CI Limit

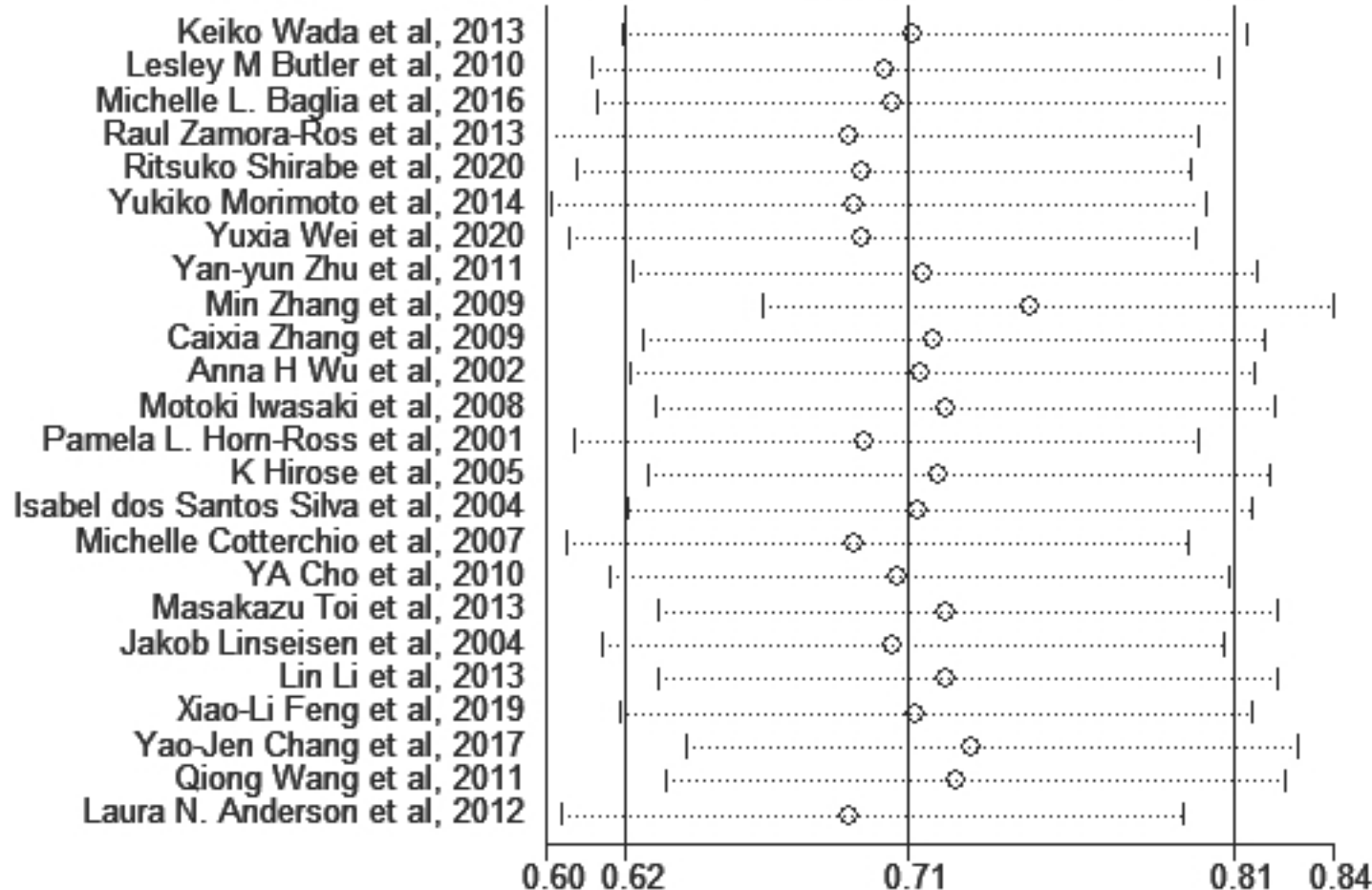

Supplement: Supplementary file 1 [file nutrients-15-02402-s001.zip › Figure S5.pdf]
